# The Ethnography of Caring Networks: Disentangling a Governance Order *In-the-Making*

**DOI:** 10.5334/ijic.10221

**Published:** 2025-11-19

**Authors:** Oemar van der Woerd

**Affiliations:** 1 Department of Healthcare Governance and Erasmus Centre for Healthcare Management, Erasmus School of Health Policy & Management, Erasmus University Rotterdam, The Netherlands

**Keywords:** network governance, healthcare management, ethnography, interpretative research

## Abstract

This thesis shifts attention from networks as well-demarcated governance structures to seeing networks as dynamic and emerging social phenomena. Drawing on ethnographic fieldwork in Dutch older person and hospital care, it explores how networking unfolds in everyday governance actions and interactions of affected actors, and with which consequences for their role and work. This thesis calls for a recalibration of network thinking, highlighting the multiple, ongoing, place-based, multi-layered, and multi-purpose nature of networking. Rather than romanticizing network governance, this thesis offers a critical-pragmatist perspective, inviting a ‘romantic-realist’ engagement with the lived messiness of networks as a governance order-in-the-making amidst healthcare reforms.

## Introduction

Creating and maintaining networks is increasingly seen as a solution to pressing healthcare issues like increasingly older person populations and workforce shortages. Yet, networks require the reconfiguration of entrenched professional, organizational, administrative, geographical, and institutional boundaries, reconsidering current working patterns. This re-puzzling of healthcare governance is at the heart of this empirically-grounded thesis. It shifts attention from networks as well-demarcated governance structures to seeing networks as dynamic and emerging social phenomena.

This thesis signals an empirical deficit within network scholarship for ‘everyday governance’ in a multi-network context—that is, an approach that seeks to capture the enactment of grand narratives of governance by actors in specific situations [1]. This deficit can be attributed to two main assumptions: (i) networks are more-or-less placeless and context-free, and (ii) networks are given and bounded entities. Both assumptions seemingly focus on formal aspects of networks. The conceptualization of *networking* leads to the research question: *How does networking unfold in the everyday governance actions and interactions of affected actors, and with which consequences does this come for their role and work?* Caring networks—as the central study object—can be understood as formalized or informal networked governance arrangements in the field of healthcare that consist of nodes and ties between multiple actors in attempts to (re)organize care provision for citizen populations, ranging from healthcare organizations and professionals, to policymakers and regulators.

The selected cases of networking in Dutch older person and hospital care are diverse in terms of geographical place, origin and (institutional) history. The empirical chapters examine (i) how actors work with networks and (ii) how a network logic is constructed. The first part analyzes how hospital managers navigate through a ‘network of collaborations’ (*Chapter 2*), the meanings of network platforms for actors involved (*Chapter 3*), and network-building in practice (*Chapter 4*). The second part focuses on mediating policy figures and their network engagement (*Chapter 5*), and how regions are ‘transformed’ into a governance object for organizing older person care (*Chapter 6*).

## Methodology

Through a multi-sited ethnography, I tried to unravel how a policy discourse that relies on networks unfolds and is enacted through actors’ actions on the ground [1]. The fieldwork covers multiple rounds of in-depth interviews, (non-)participant observations, and document analysis. I followed an abductive logic of inquiry and analysis, iteratively moving back and forth between empirical data and theoretical work about network and collaborative governance. Data consists of observational reports, interview transcripts, and policy and organizational documents. The analytical focus was narrowed down to the interactions among affected actors (e.g. healthcare managers, policymakers, policy advisors, physicians, nurse practitioners, and national authorities). The combination of ethnographic methods led to rich narratives about networking, bolstering an iterative process of triangulation to validate findings.

## Findings

The cases of networking in older person and hospital care researched put forward the multiple, ongoing, place-based, multi-layered, and multi-purpose nature of networking.

The *multiplicity* of networks shows that networking is no standalone activity within the boundaries of a network, but is tied with nodes of multiple networks [2]. Healthcare managers and professionals relate to many overlapping and conflicting interests, purposes, ambitions, laws and regulations, and emotions simultaneously.

*Ongoing* entails that networking has no clear stop, but requires continuous work while navigating organizational, epistemic, and normative ambiguities that have to be processed over and over again [3]. It comes with a ‘web of interests’ (i.e. personal, organizational, regional, societal, external stakeholders) that requires recurrent alignment work [2].

*Place-based* means that networking cannot be decoupled from the sociocultural, institutional, and geographical context in which it is aimed to have an effect [3]. Mediating policy figures become important yet hidden actors around network formation. The practices through which strategizing and legitimizing occurs in relation to networks encompasses more than constructing a network entity [5].

*Multi-layered* means that networking is embedded in underlying dynamics like professional-management relations and interactions, but also ties into broader governance structures [5]. It encompasses both inward and outward work (e.g. moving in and between networks and the organization) to adapt to (changes in) the regulatory environment with conflicting accountability schemes [2].

*Multi-purpose* encompasses the various ways that purposes come into being through networking, underscoring the sensemaking possibilities for actors [4]. Network purposes are not fixed in time, but subject to change. Networking is thus not static, but dynamic—full of ambiguities and relational processes in which interactions and structures are made and unmade [1].

## Implications for integrated care and future research

The increasing emphasis on networking as a practice of care requires healthcare professionals and managers to live with and endure the imperfections of networking, but also to tinker with them further and become proficient in networking.

A looming danger for policymakers is to simplify the everyday consequences of networking. Learning from field-level narratives of networking may enable policymakers to be aware of the particularities of their policy activities, broadening the value systems that are used in networked policies.

Networking is no power-free practice in which actor perspectives can be harmoniously woven together into one (regional) perspective. Networking does not only ‘integrate’ things, but also comes with exclusion. Such an understanding may prevent networking from becoming primarily a process for elite societal or professional groups. Who stays behind in networking? How to organize and account for (citizen) representation and participation? Sensitivity towards these questions is necessary to make networking a diverse and democratic practice of care.

Further exploration of the dark sides of a multi-network context is warranted as networking is not merely attractive for actors. It may reproduce differences in terms of network capital or dominant interests. How to cope with structural disparities in terms of network capital between the places where networks take shape? How does networking reconfigure interdependencies among actors? Networking may evoke the redistribution of professional tasks, which may have an impact on how formal and informal caregivers, as well as citizens, clients, and patients construct and frame ‘good care’.

## Limitations

The particularities and complexities of the Dutch healthcare system make the study of networking in this thesis situated and thus difficult to generalize to other settings. Situated findings are theoretically generalizable as they apply to wider debates on networked policies, and how this is enacted in actors’ lifeworld’s. Although the cases are not systematically compared, I tried to relate the case contributions to each other by searching for underlying mechanisms, i.e. a certain type of work. Furthermore, capturing what actors do might be seen as a flat ontology. What I tried in this thesis was to critically reflect upon the actions of many interacting actors, providing analytical depth into the ethnographic findings. This thesis tried to analyze a governance order in-the-making, but the thesis itself is also not final. My hope is that it will contribute to a more nuanced public debate about the prophecy of (caring) networks in changing societies.
